# Peripheral Stimulation of the Saphenous and Superior Lateral Genicular Nerves for Chronic Knee Pain

**DOI:** 10.7759/cureus.14753

**Published:** 2021-04-29

**Authors:** Jamal Hasoon, Ahish Chitneni, Ivan Urits, Omar Viswanath, Alan D Kaye

**Affiliations:** 1 Department of Anesthesia, Critical Care and Pain Medicine, Beth Israel Deaconess Medical Center and Harvard Medical School, Boston, USA; 2 Department of Anesthesiology, A.T. Still University School of Medicine, Mesa, USA; 3 Department of Anesthesia and Pain Management, Valley Anesthesiology and Pain Consultants, Phoenix, USA; 4 Department of Anesthesia and Pain Management, Louisiana State University Health Sciences Center, Shreveport, USA

**Keywords:** knee pain, chronic pain, neuromodulation, peripheral nerve stimulation, arthritis

## Abstract

Chronic knee pain continues to cause increasing levels of functional deficits, mobility issues, and decreased quality of life in the United States. Initial treatment for knee pain consists of physical therapy, weight loss, medication management, injections, and radiofrequency ablation (RFA). Definitive treatment usually requires surgical management. Peripheral nerve stimulation (PNS) has been effective in the treatment of a variety of chronic pain conditions including the treatment of postoperative pain related to knee surgery. We describe the case of a patient who refused operative management as well as RFA of the genicular nerves and obtained significant pain relief from PNS of the superior lateral genicular nerve and the saphenous nerve for severe knee pain caused by osteoarthritis.

## Introduction

Chronic knee pain continues to cause increasing levels of functional deficits, mobility issues, and decreased quality of life in adult patients in the United States. In fact, knee pain has been shown to affect over 25% of adults with a prevalence that has increased 65% over the last two decades [1]. Data have shown that approximately 18 million patients visit a physician each year due to knee pain [2]. Initial treatment options for knee pain in adults typically consists of conservative management such as physical therapy and weight loss. Active management techniques such as stretching and various other exercise programs are also recommended as first-line intervention. In addition, medication management with acetaminophen and nonsteroidal anti-inflammatory drugs (NSAIDs) has also been shown to result in pain relief and is considered first-line pharmacological treatment [3]. In many cases, conservative therapy and pharmacological options are unable to resolve knee pain, and further intervention may become necessary.

Commonly utilized interventions by physicians include the administration of intra-articular corticosteroid injections. Past research has concluded that corticosteroid injections for the management of knee pain in osteoarthritis is variable and typically only provides pain relief for a few weeks. In addition, the use of corticosteroid injections is recommended to be limited to once every three months to decrease overall steroid use in patients [4]. Other forms of knee pain management include genicular nerve blocks (GNB) and radiofrequency ablation (RFA). In certain cases, postoperative knee pain that occurs after total knee replacement has been shown to be reduced using GNB [5]. Research has also shown that there is potential benefit with RFA of the genicular nerves to relieve chronic knee osteoarthritis pain [6]. Despite the use of these therapies, many patients may not respond to these treatment options.

Peripheral nerve stimulation (PNS) is a pain management procedure that uses electrical currents to target specific nerves. Research has shown that PNS is effective in the treatment of chronic pain conditions such as trigeminal neuralgia, cluster headaches, post-herpetic neuralgia, and complex regional pain syndrome [7]. Typically, PNS consists of an electrode that is implanted next to the nerve of choice, which can then be stimulated with the hope of achieving pain relief. Past studies have been conducted on the use of PNS for postoperative pain relief following knee surgery. However, research on the use of PNS for nonsurgical candidates or patients refusing surgery is yet to be conducted.

We present the use of PNS targeting the superior lateral genicular nerve and the saphenous nerve for a patient with severe knee pain from osteoarthritis who refused operative management as well as RFA.

## Case presentation

The patient was a 58-year-old male with a history of chronic pain related to osteoarthritis of the right knee. The patient noted that his pain was dull and aching in nature and significantly impacted his ability to work and his quality of life. He noted that the pain was not relieved with medication management, including acetaminophen, NSAIDs, membrane stabilizers, and high-dose opioids. He reported a pain score of 10/10 intensity on a numerical rating scale. The patient refused total knee replacement surgery and desired other modalities to control his pain. He underwent intra-articular steroid and viscosupplementation injections with minimal improvement of his pain. The patient was offered diagnostic GNB and subsequent RFA versus a temporary 60-day PNS system (SPRINT PNS System [SPR Therapeutics, Inc., Cleveland, OH]). The patient decided to pursue PNS for his chronic knee pain.

**Figure 1:**
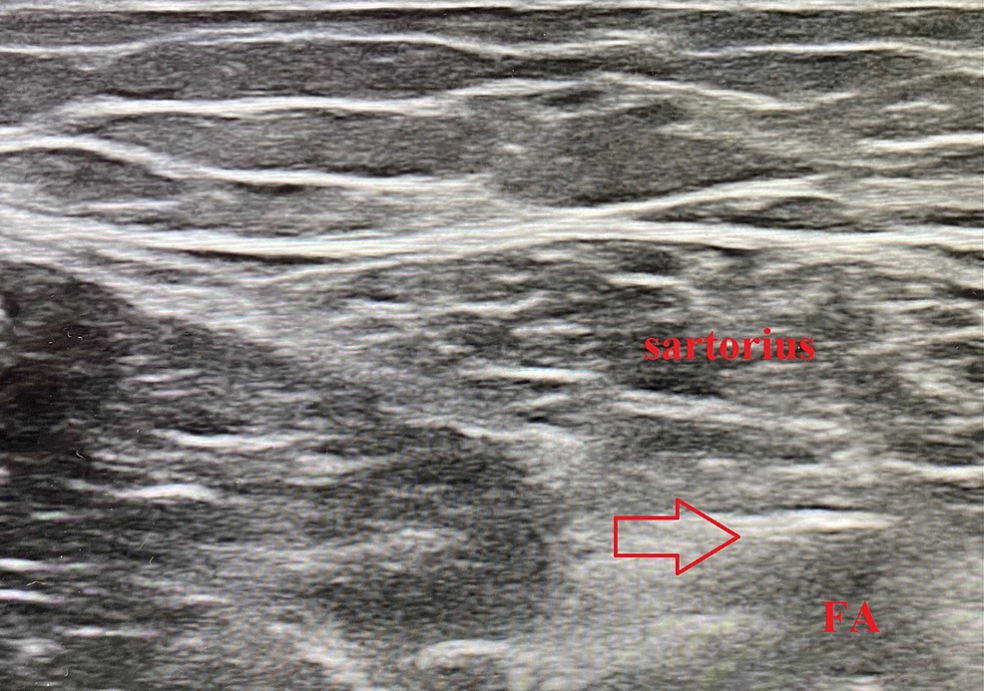
Ultrasound imaging of the adductor canal. Ultrasound was used to identify the sartorius muscle and the adductor canal. The adductor canal contains the femoral artery, femoral vein, and saphenous nerve. The needle was placed in the region near the red arrow which provided adequate stimulation of the saphenous nerve

The patient was positioned supine and his right leg was prepped and draped in a sterile fashion. Utilizing a 12 MHz linear ultrasound transducer, the saphenous nerve was identified in the adductor canal near the sartorius muscle [Figure 1]. The saphenous nerve was targeted as it is a sensory branch of the femoral nerve that supplies sensory innervation to the medial aspect of the knee as well as infrapatellar branches to the knee joint. The PNS lead was placed roughly 0.5 cm from the saphenous nerve and secured once the patient reported a comfortable paresthesia over the medial portion of the knee.

**Figure 2:**
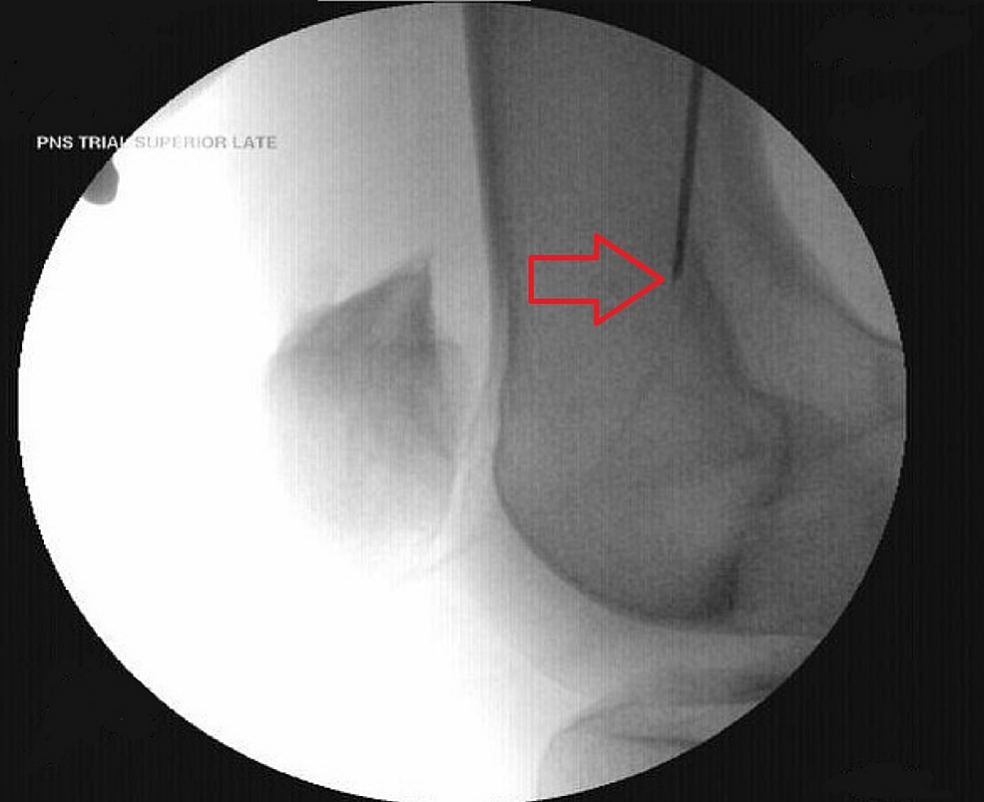
Superior lateral genicular lead placement with fluoroscopic guidance. Fluoroscopy was used to place the lead in the region of the superior lateral genicular nerve. The red arrow is pointing toward the final position of the introducing needle. The needle was repositioned until optimal paresthesia coverage was obtained

Next, the superior lateral genicular nerve was targeted utilizing fluoroscopy. A lateral view was obtained, and the entry point marked slightly cephalad to the lateral epicondyle. The introducer needle was entered and advanced in a caudal direction until it contacted the periosteum [Figure 2]. The lead was repositioned until a comfortable paresthesia was reported over the lateral aspect of the knee. The leads were then secured without complications.

The patient reported substantial improvement shortly after the procedure nearing 100% pain relief. He significantly increased his overall activity levels noting he was able to resume his normal work requirements. He was also able to move furniture into a new home and enjoy outdoor activities. The patient noted the procedure drastically increased his functionality and quality of life. Unfortunately, the patient noted that one of the leads became dislodged due to his increase in activity level, and he removed both leads before they could be salvaged one month into his trial. Given his drastic improvement, the patient is currently considering retrial with a temporary 60-day PNS system to see if this provides longer relief following the full 60-day therapy or pursuing a permanent PNS system.

## Discussion

Our literature search has shown that much of the use of PNS for knee pain has been for postoperative pain relief [8]. In addition, no studies or case reports on the use of PNS for knee pain among patients refusing knee surgery have been conducted or reported. In some cases, RFA has been performed for knee pain. A double-blind, randomized controlled trial conducted by Choi et al. studied the use of radiofrequency treatment for chronic knee osteoarthritis pain. The results showed that the radiofrequency group reported a 50% reduction of knee pain during measurements taken at one, four, and twelve weeks post-procedure [6]. Another study conducted by McCormick et al. used cooled radiofrequency ablation (C-RFA) of the genicular nerves to treat patients with severe chronic osteoarthritis pain. In this study, C-RFA was performed on 33 patients, and a six-month outcome follow-up was conducted. In this study, only 35% of procedures resulted in 50% or greater reduction in pain, and only 19% of procedures resulted in total pain relief [9]. Although C-RFA was able to provide pain relief over the course of six months, less than one half of the procedures resulted in 50% or greater pain reduction. In addition, a study conducted by Ikeuchi et al. evaluated the use of percutaneous radiofrequency treatment for patients with osteoarthritis and refractory anteromedial knee pain [10]. Patients who underwent RFA showed significantly decreased pain scores compared to the control group. Despite the significance between the groups seen in the study, the percentage of responders in the RFA group was 50% at four weeks, 30% at 12 weeks, and 0% at six months. The lack of data showing pain reduction in the entire group that received RFA treatment shows that alternate methods of pain relief may be a more viable option.

Several studies have also been conducted on the use of PNS for knee pain. A systematic review conducted by Lin et al. reviewed studies conducted on the use of PNS. Results identified six studies, four of which used PNS for postoperative knee pain and two studies which used PNS for neuropathic pain [11]. One of the studies identified in the review was conducted by Ilfred et al. which assessed the use of percutaneous PNS for postoperative pain after total knee arthroplasty on seven subjects [8]. Results of the study showed that six of the seven subjects had an average pain score of <4 after first two weeks after treatment with PNS, and after 12 weeks had improvements in the six-minute walk test and Western Ontario and McMaster Universities Osteoarthritis Index scores. In addition to decreased pain scores, all patients discontinued opioid use within one week post-surgery due to the lack of postoperative knee pain.

These studies demonstrate that PNS is a feasible option for postoperative pain relief following knee surgery and should be considered as a method of pain management to decrease opioid usage and pain level. However, our case demonstrates that PNS may provide some benefit for patients who are unable or unwilling to undergo total knee arthroplasty.

## Conclusions

Although studies have been conducted on the use of PNS for postoperative analgesia, the literature on the use of PNS for patients refusing surgery is limited. To our knowledge, we discuss the first use of PNS targeting the superior lateral genicular nerve and the saphenous nerve for a patient with severe knee pain from osteoarthritis who refused operative management as well as RFA. Our results are encouraging, and we believe that PNS may be a viable option for patients who do not qualify for surgical intervention, refuse surgery, or have failed other conservative and pharmacological methods of treatment. However, further studies are needed to confirm the findings of this single case report.
